# Supplementary material for: Are carbon emissions trading and green financial instruments synergistic? -Comprehensive quantitative research based on content analysis
Source: PLoS One. 2024 Mar 7;19(3):e0298601. doi: 10.1371/journal.pone.0298601 (PMC10919601; doi:10.1371/journal.pone.0298601)
Supplement: S1 Data — (ZIP) [file pone.0298601.s001.zip › Data/地级市Z_F工作报告.zip!/╔╜╬≈/┴┘╖┌╩╨/└┤╫╘╣1⁄2╓┌║┼í╛┬φ┐╦╩2╛▌═°í┐_2015.docx]

各位代表：

现在，我代表市人民政府向大会报告工作，请予审议，并请市政协委员和其他列席人员提出意见。

一、2014年工作回顾

2014年，我们深入贯彻党的十八大、十八届三中、四中全会和习近平总书记系列重要讲话精神，认真落实省委、省政府的工作部署，扎实开展“四个年”活动，大力实施“六大惠民工程”，统筹推进稳增长、促改革、调结构、惠民生，全市各项工作稳中有为、稳中有进，在全面建成小康社会的道路上迈出了坚实的步伐。

（一）千方百计稳增长，经济保持平稳发展。面对主要产品价格下跌、企业效益下滑、金融运行偏紧，经济下行压力加大的严峻形势，我们坚持把稳增长作为首要任务，努力实现经济平稳运行。

把稳工业保运行作为头等大事。认真落实国家、省一系列稳增长政策，出台了“工业经济运行8条”、“促进市内工业产品销售15条”等措施。强化经济运行监测和督导服务，实施“一业一策、一企一策”精准帮扶，分别召开煤炭、钢铁、焦化、电力等主导产业运行会议，帮助企业解决实际困难和问题，协调落实贷款22.7亿元；以晋南钢铁贸易公司为代表，推动企业采取各种形式联合发展，引导企业抱团取暖共渡难关。积极扶持小微企业发展，创办小微企业1880户，新增个体工商户14033户，培育“小升规”企业24户、“小巨人”企业11家。

把扩投资增效益作为关键举措。实施省市县重点工程903项，完成投资1304亿元，在“六位一体”全省考核中，夺得1项第一、1项第二、5项第三、3项第四。重点推进“百里汾河生态经济带”建设，经济带总体规划纳入《晋陕豫黄河金三角区域合作发展规划》，获国务院批复，6个专项规划通过国家级专家评审；投资43亿元的汾河河道生态治理工程全面竣工，投资42亿元的滨河东路贯通工程基本竣工，投资38亿元的涝河生态环境综合治理初见成效，投资32亿元的尧都尧庙片区、投资10亿元的洪洞甘亭“六村连片”等11个“两区同建”工程扎实推进。

把抓安全促稳定作为重要保障。认真贯彻新《安全生产法》，扎实开展“知责、履责”活动，深入开展安全生产大检查，认真组织“六打六治打非治违”专项行动，切实加强对煤矿、非煤矿山、危险化学品、道路交通、油气管道、粉尘防爆、食品药品、消防等重点领域的安全监管，全年各类生产经营性安全事故起数和死亡人数完成省下达控制指标，没有发生重特大事故，安全生产形势持续稳定好转。推进社会治理创新，组织开展“百日双百案”群众信访诉求化解专项活动，有序化解社会矛盾，维护良好的社会秩序，为经济发展营造了安全稳定的社会环境。

2014年，全市生产总值完成1213亿元，同比增长4.6%；规模以上工业增加值完成467亿元，增长3%；固定资产投资完成1229亿元，增长18.6%；社会消费品零售总额完成530亿元，增长11.3%；公共财政收入完成118亿元，与上年持平；城镇居民人均可支配收入23610元，增长8.2%；农村居民人均可支配收入8755元，增长11.4%。

（二）蹄疾步稳促改革，发展活力不断增强。我们以转型综改试验区建设为统领，全面实施“16112”年度计划，全市确定的10大项26条改革举措稳步推进。

深入推进行政审批制度改革。着力实施简政放权，承接省政府审批事项30项，下放县级行政审批事项14项，取消行政审批事项4项，取消行政事业性收费19项。编制“权力清单”、“责任清单”，市本级行政审批事项减少到102项，精简了39%。市政务服务中心投入使用，实行“一委一办三中心”的管理体制，全面推行“两集中、两到位”；市级公共资源交易平台建成运行，完成各种交易事项548场次，总成交额近30亿元；“市长热线”正式开通，公共服务质量进一步提高。

扎实推动重点领域改革。清理规范涉煤收费项目，推进煤炭资源税从价计征改革，实施煤炭公路销售体制改革，撤销煤焦站点79个，吨煤降低成本60元，为我市煤炭企业减负36亿元。在全省率先开展了非煤矿山收费清理规范工作。市县两级政府机构改革、工商登记制度改革、国有企业改革、财税体制改革、开发区扩区改革、招商机制改革等有序推进。

大力实施创新驱动发展战略。制定实施《国家创新驱动发展战略临汾行动计划》和《临汾市低碳创新行动计划》，全市高新技术企业发展到28家，有效专利拥有量达到271件，实施国家和省级科技项目46项、市级53项。“千人百县”服务基层活动扎实推进。

澳坤生物公司在“新三板”挂牌上市，实现了我市地方企业在全国股权转让系统上市“零的突破”；我市在省股权交易中心挂牌的企业达到100家。

（三）坚定不移调结构，产业转型明显加快。牢牢抓住结构调整这条主线，全市三次产业比例从7.1∶59.9∶33调整优化为7.8∶54.4∶37.8。

现代农业加快发展。粮食总产27.6亿公斤，再创历史新高。“一村一品”“一县一业”和“四个百万亩”建设扎实推进，新发展设施蔬菜3.9万亩、水果11万亩、干果10万亩、中药材26万亩，市级以上标准化规模健康养殖企业达到79个，农业机械化率提高到70.5%。10大现代农业园区规模发展。全市认定家庭农场950个，新注册农民专业合作社8728家，农业产业化龙头企业达到394家，农产品加工销售收入达到70亿元。按照精准扶贫的要求，完成了贫困村和贫困户的建档立卡工作；实施产业扶贫开发项目35个，完成投资28.2亿元；开展住村联户、片区开发、易地扶贫搬迁等工作，实现脱贫6.1万人。汾西、大宁、吉县成为国家首批光伏扶贫试点县。

工业经济提质增效。加快煤矿基本建设步伐，新竣工矿井21座，全年原煤产量5069万吨。实施焦化、冶金、电力等传统产业改造项目13个，完成投资40亿元。实施装备制造、新能源、新材料等新兴产业项目74个，完成投资262.7亿元，非煤产业增加值比重达到50.4%。积极化解过剩产能，淘汰落后焦化产能408万吨、炼钢产能260万吨、炼铁产能33万吨。

第三产业发展提速。商贸服务、电子商务、现代物流、文化创意、旅游休闲等产业蓬勃发展，奥特莱斯芭蕾雨、新百汇、生龙国际等城市商业综合体项目加快实施。侯马开发区国家电子商务示范基地入驻电商企业42家；临汾开发区电子商务产业孵化园入驻企业20家。山西国际陆港等物流园区建设持续推进。曲沃晋国青铜玉器文化产业园、侯马晋园文化城等一批重点文化产业项目加快实施。汾河公园、侯马彭真故居、蒲县东岳庙等3个景区成功创建国家4A级旅游景区，开展了2014临汾金秋游（北京）推介会等旅游宣传活动，全市旅游总收入达到242亿元，增长24%。

生态环境明显改善。狠抓节能减排，全市万元地区生产总值综合能耗及化学需氧量、氨氮等指标超额完成省定任务。成功跻身国家30个节能减排财政政策综合示范城市，成为全省唯一入选的城市。尧都区城东老工业区搬迁改造纳入国家试点，浮山县入选国家资源综合利用“双百工程”示范基地，西山7县成为国家主体功能区建设试点。实施大气环境、水体质量、城乡清洁、生态治理和交通秩序五大提升工程，淘汰黄标车及老旧车3.4万辆；市区改造天然气5万户，空气综合质量指数下降12.7%，PM2.5浓度下降14.9%，二级以上天数240天，增加73天。市污水处理厂改扩建一期进入调试阶段。全市主要河流地表水水质持续改善。全市完成营造林46万亩。市区新增绿化面积25.3万平方米，成功创建省级园林城市。

（四）尽心竭力惠民生，持续改善人民生活。

“六大惠民工程”成果丰硕。2014年是我市实施“六大惠民工程”的第一年，我们完善政策，加大投入，突出重点，有序推进。

实施学前教育优质普惠工程，出台了《城镇小区配套幼儿园规划建设与使用管理办法》，新改扩建幼儿园63所，取缔不达标幼儿园336所。实施乡村医疗服务提质工程，新农合人均筹资标准由340元提高到390元，其中政府补助由280元提高到320元，参合率达99.3%，重大疾病医疗保障范围增加到23种，新农合大病保险全面推开。实施农业强基工程，持续推进“大水网”涉临工程，完成洪洞曲亭水库修复和11座中小型病险水库治理，改造中低产田32.5万亩。实施饮水安全工程，市区新建城市供水网8.3公里，改建3.5公里，各县市新建城市供水网47.6公里，改建29.5公里；改善和提高了5万农村人口的饮水安全标准。

实施城市综合整治工程，开展市容专项整治活动，清理马路堆放、马路经营、马路加工25000余处，规范早市、夜市340处；市区新建公交站点42个，新增公交车78辆、线路里程54公里。实施就业创业援助工程，全市城镇新增就业5.19万人，转移农村劳动力5.26万人，城镇登记失业率为2.9%。

城乡面貌明显改善。全年建设高速公路182公里，完成国省干线公路改造52公里，新改建农村公路270公里。大西高铁、中南铁路、霍永高速东段和西段一期建成通车，吉河高速完成路基工程，霍永高速西段二期全面启动。临汾机场进入通航前的验收阶段。城西客运站基本建成。编制了《市区地下空间利用规划》、《南部机场片区控制性详细规划》等重点规划。在财力紧张的情况下，采取PPP模式、“334”投融资方式等多种办法，投资16亿元，新建改造市区道路11条，其中，五一路贯通、秦蜀路南延拓宽改造、景观大道、滨河西路北延、新高中南路等道路竣工通车，二中路、规划三街南段、中大街南段等工程正在建设。市规划展览馆建成投用，市博物馆、图书馆正在建设，市奥体中心、临汾学院、山西师大搬迁等项目正在推进。城中村改造进展顺利，改善农村人居环境“四大工程”扎实推进，农村“五件实事”全部完成。

民生事业协调发展。我们坚持真情实意惠民生，民生支出占到全年财政总支出的84.4%。完成5个县薄弱学校改造；培养名师358名、名校37所、名校长39人，教育教学质量稳步提升。市人民医院新院区顺利开诊；县级公立医院改革实现全覆盖。全市人口自然增长率控制在5.4‰。完成了第四批国家级非遗项目申报工作，有3个项目成功申报国家级名录。全市有线电视村村通覆盖率达81%。成功举办了中国?临汾2014国际名校赛艇挑战赛。《临汾市志》正式出版。社会保障不断加强，7项社保参保总人数达到473万人。新开工保障性住房18682套，基本建成16044套。规范机关事业单位津、补贴，最低工资标准月人均增加160元，企业工资基准线增长13%，企业退休人员养老金实现“十连涨”。我市被确定为国家信息惠民试点城市。

与此同时，积极推进军民融合发展，国防动员和国防后备力量建设得到加强。民族宗教、外事侨务、老龄、残疾人等事业进一步加强，人防、统计、气象、水文、档案、地震、防灾减灾等工作取得新进展。

我们推进民主法制建设，认真执行人大及其常委会的决议、决定，积极支持人民政协履行职能，共承办人大代表建议、意见110件，政协委员提案184件、意见61件。支持各民主党派、工商联、无党派人士参政议政，支持工会、共青团、妇联等人民团体发挥作用。深入开展“六五”普法，公民法治意识进一步增强。

我们扎实开展党的群众路线教育实践活动和学习讨论落实活动，严格执行中央“八项规定”和国务院“约法三章”，坚决反对“四风”，着力解决人民群众反映强烈的突出问题。会议、文件分别削减15.3%和7.7%，“三公”经费支出同比下降34.7%。不断加强党风廉政建设，认真落实“一岗双责”，加大对政府权力运行的监督力度，严肃查处发生在行政审批、公共资源交易中的违纪案件，切实加强审计监督，政府廉政建设得到进一步加强。

各位代表，过去一年的成绩来之不易，这是省委、省政府和市委正确领导的结果，是市人大、市政协和社会各界监督支持的结果，是全市人民凝心聚力、顽强拼搏的结果。在此，我代表市人民政府，向全市人民，向驻临部队、武警官兵和公安民警，向各民主党派、工商联、人民团体，向所有关心、支持、参与临汾改革发展的各界人士，表示崇高的敬意和衷心的感谢！在总结成绩的同时，我们也清醒地认识到存在的问题，主要是：受经济下行影响，虽然年初确定的各项约束性指标全面完成，但地区生产总值、规模以上工业增加值等预期性目标未能实现；战略性新兴产业发展不够，科技创新能力不强，转型升级任务艰巨；财政增收放缓，刚性支出需求加大；生态环境仍然脆弱，安全生产基础还不牢固；农民增收难度加大，扶贫开发任务艰巨；公共服务水平仍需提高，民生社会事业仍需进一步加强；政府职能有待进一步转变，“四风”问题尚未根绝，政务环境还需优化，庸政懒政怠政现象仍然存在，等等。对于这些问题，我们要保持清醒头脑，坚持勇于担当，采取有力措施，认真加以解决。

二、2015年工作安排

今年是按照“四个全面”战略布局开拓前行的重要一年，是决胜“十二五”、谋划“十三五”的关键之年，也是全面推进“六权治本”“六大发展”的开局之年，改革发展稳定的任务艰巨繁重。

今年国内外经济形势依然十分严峻复杂，经济下行压力持续加大，这必将给我市经济运行、财政收支及各项事业带来更大困难。但是，我们必须看到面临的有利条件和难得机遇，国家实施积极的财政政策，推动“一带一路”建设，加快京津冀协同发展，这些都与我们的主导产业密切相关；我省加强党风廉政建设和反腐败斗争，加快法治山西建设，将为经济社会发展提供风清气正的良好环境，省委提出“六大发展”的战略思路，掀开了山西发展的新篇章，必将迎来加快发展的新热潮；特别是经过近几年的努力，我市交通优势、区位优势、环境优势进一步显现，“百里汾河生态经济带”引领作用持续增强，我市将迎来要素汇聚、潜力迸发的发展机遇期。我们要认识新常态，适应新常态，引领新常态，在逆境当中看到希望，在压力之下坚定信心，在困难面前抢抓机遇，始终坚持发展是第一要务，坚定不移地推进临汾加快发展；始终坚持深化改革、扩大开放，以创新创造激发活力，增强动能；始终坚持保障和改善民生，以增进民生福祉为根本目的，让人民群众得到实实在在的好处。

今年政府工作的总体思路是：深入贯彻党的十八大、十八届三中、四中全会和十二届全国人大三次会议精神，按照“四个全面”战略布局，以习近平总书记系列重要讲话精神为根本指针，认真落实省委、省政府和市委的决策部署，主动适应经济发展新常态，坚持稳中求进总基调，牢牢把握稳增长和调结构的平衡点，狠抓“六权治本”，推进“六大发展”，全面深化改革，不断扩大开放，突出创新驱动，保障改善民生，实现经济持续健康发展和社会和谐稳定，不断开创弊革风清、富民强市新局面。

今年经济社会发展的主要预期性目标是：地区生产总值增长6%，规模以上工业增加值增长7%，全社会固定资产投资增长16%，社会消费品零售总额增长10%，公共财政预算收入增长6%，城镇居民人均可支配收入增长8%，农村居民人均可支配收入增长9%，居民消费价格涨幅控制在3%以内。约束性指标是：万元地区生产总值能耗下降4.29%，二氧化硫、化学需氧量、氨氮、氮氧化物排放量分别下降3.77%、2.63%、3.42%和3.97%。

围绕上述目标，今年要重点做好九方面的工作：

（一）坚持稳中求进，促进经济平稳健康发展把握关键稳增长。我们要坚定信心，迎难而上，抓住关键环节，解决突出问题，采取有力措施，努力实现经济企稳回升。抓基础、保均衡，紧紧抓住基础产业、重点企业，坚持市县领导包联企业，建立常态生产调度机制，实行一企一策、精准服务，强化经济运行监测分析，保持经济均衡发展。全市规模以上企业煤炭产量达到6000万吨、焦炭产量达到1800万吨、生铁达到1100万吨、钢材达到1200万吨、发电量达到210亿度。

抓资金、保运行，认真落实省政府减轻企业负担60条措施，实施好我市确定的65条减负措施；搞好银企沟通对接，争取金融机构扩大信贷投放，有效缓解企业融资难、融资贵问题；设立中小企业“过桥还贷”资金，帮助企业渡过难关。抓销售、保市场，强化煤电油运等企业生产要素保障，促进主要产品市场销售，组织企业积极开拓市场，巩固扩大产品话语权和市场份额。抓合作、促共赢，引导企业抱团取暖，支持同类企业之间、上下游企业之间，采取组建企业集团、企业联盟等多种方式加强联合，提高抵御市场风险的能力。

抓好项目稳增长。把投资作为稳增长的强大引擎，围绕省政府确定的“四个方面、十大领域”投资方向，扎实开展“项目提质增效年”活动，实行重大项目“双包双联”责任制，健全项目调度推进机制，破解项目用地、资金等要素制约，“六位一体”统筹推进项目建设。全年项目储备动态保持在12000亿元，签约完成2000亿元，落地完成1580亿元，开工完成1350亿元，建设完成1130亿元，投产完成1260亿元，全社会固定资产投资完成1426亿元。实施总投资3834亿元、当年投资1260亿元的922项省市县重点监测项目。铁路投资42.5亿元，加快推进张台铁路、中南铁路战略装车点等13项铁路项目建设；公路投资83.6亿元，吉河高速年底前竣工，霍永高速西段二期、长临高速工程全面开工建设，集中连片特困地区交通扶贫项目完成146公里；水利投资41.2亿元；新兴产业项目投资337亿元；城乡人居环境改善投资447亿元。

招商引资稳增长。进一步加大招商引资力度，努力开工一批、争取一批、签约一批、招商一批大项目、好项目。优化服务促招商，狠刹“吃拿卡要”的歪风，努力营造良好的政务环境，倡导形成高效优质的服务理念，全面推动精准招商、感情招商、产业招商、要素招商、园区招商。突出重点促招商，紧盯长三角、珠三角、京津冀等重点招商地区，围绕“十大领域”、七大产业等重点招商范围，吸引世界五百强、中国五百强等重点企业，加快承接沿海发达地区产业转移。借势借力促招商，抓住国家采取积极的财政政策、实施“一带一路”建设、京津冀协同发展等重大的政策机遇，发挥我市百里汾河生态经济带、黄河金三角、节能减排试点城市、方略综合物流保税中心等重要平台的作用，积极参与跨国公司入晋项目推介上海专场活动、晋粤交流合作广州项目对接会、央企入晋北京项目对接活动等30项投资促进活动以及乌洽会、投洽会、西博会、中博会等招商活动，自主举办各类招商引资洽谈会，确保招商引资取得实效。

龙头带动稳增长。进一步加大“百里汾河生态经济带”建设力度，按照全省《汾河流域生态修复规划纲要》，加强汾河流域生态修复，保护汾河生态文明。认真实施“经济带”《总体规划》和6个《专项规划》，启动总投资1892亿元，涉及生态环保、新型工业、现代农业、现代服务业、基础设施和民生类，共251项“经济带”项目建设。

进一步完善路网、水网、电网、通讯网，加快临汾民航机场通航，加快涝??河生态环境综合治理工程建设；进一步推进“经济带”内的18个工业园区、10个现代农业园区、6个物流园区和5个文化旅游园区建设，重点抓好投资64亿元的侯马北铜铜业产业园、投资30亿元的汉能柔性薄膜太阳能电池生产项目、投资102亿元的山焦年产60万吨烯烃等项目建设；加快推进“两区同建”，重点抓好洪洞天泽、襄汾丁村、侯马上阳、霍州西张垣等11个“两区同建”项目建设。

（二）做好煤与非煤两篇文章，推动产业结构优化升级大力推动“煤炭革命”。落实我市《推进煤炭产业“六型”转变实施方案》，促进煤炭产业向“市场主导型”、“清洁低碳型”、“集约高效型”、“延伸循环型”、“生态环保型”和“安全保障型”“六型”转变。加快煤矿基本建设步伐，全年地方监管矿井基本建设完成投资30亿元，新增竣工矿井27座，新建现代化矿井5座。发挥我市主焦煤优势，组建临汾焦煤集团和中国（太原）煤炭交易中心临汾办事处。提高煤炭技术创新能力，全年煤矿技术投入比不低于5%。实施我市《矿山生态环境保护与恢复治理方案》，加强矿区生态建设。

改造提升传统产业。加快非煤矿山、焦化、钢铁、电力等传统产业改造步伐。非煤矿山重点做好铁矿资源整合和复工复产工作，加快汾西其亚铝业项目建设。焦化产业抓好淘汰落后、产业聚集、链条延伸，加快推进襄汾万鑫达20万吨粗苯精制、古县利达LNG等项目建设。钢铁产业抓好产品优化、整合重组，做强做大晋南钢铁集团，重点实施新金山100万吨高速线材、通才工贸120万吨球团等项目，加快推进翼钢重组，着力优化品种结构，逐步开发优特钢。电力产业围绕装备提升、超低排放，重点推进洪洞晋能、蒲县宏源、古县西山煤电、翼城阳煤等低热值煤发电项目。

培育壮大新兴产业。积极发展装备制造业，以煤炭机械、汽车零部件、工程机械配件、家电配件为重点，逐步向高端制造延伸，抓好好利阀门、东方恒略精密铸造、沃特玛新能源汽车核心零部件产业基地、德讯电梯等项目建设。积极发展新材料产业，推动新型建筑材料、高端金属结构材料、特种金属功能材料发展，重点推进纳海鸣建材4万吨岩棉板、浮山晋盛新型建材60万平方米微晶玻璃板材等项目建设。积极发展新能源产业，重点实施永和煤层气开发利用、侯马志盛150兆瓦太阳能电池组件等项目。积极发展食品医药产业，加快食品医药企业技术创新、精深加工和品牌建设，重点推进阳煤顶吉食品10万吨苹果深加工二期、侯马开发区医疗器械仓储物流中心等项目建设。

加快发展现代服务业。旅游业按照“一带五区一线十点”的布局，做好洪洞大槐树、乡宁云丘山、吉县壶口瀑布等国家5A级景区创建工作，加大旅游宣传营销，推进智慧旅游，全年旅游综合收入增长15%。文化产业重点培育文化产业园区和骨干文化企业，推进文化创意和设计服务与相关产业融合发展。物流业重点推进山西国际陆港、中信空港物流园等园区建设，加快发展第三方物流和多方物流。现代商贸业加快上东世纪CBD、临汾建材家居博览城等城市综合体建设，实施“电商千人百企培训工程”，推进侯马开发区国家电子商务示范基地等项目建设。实施信息惠民工程，加快构建信息惠民综合服务体系。大力发展“互联网+”，推进各行各业商业模式创新，不断催生新兴业态。

（三）深化改革扩大开放，全面增强发展动力和活力

以综改试验区建设为统领，认真落实好省定各项综改任务，按照我市三年《综改实施方案》和2015年行动计划，全面完成“5111”重大任务。深化行政审批制度改革。

按照我市《推进“六权治本”加强市政务服务中心建设实施方案》，厘清权力清单、审批清单、负面清单，切实落实“两集中、两到位”；认真做好省政府下放审批事项的承接工作，继续取消和下放一批市级行政审批项目，进一步精简审批事项，优化审批流程，提高行政效率，规范完善市公共资源交易平台建设。深化农村改革。全面铺开农村土地承包经营权确权登记颁证工作，推动农业适度规模经营，加快林权制度配套改革，探索推进农村集体产权制度、水权制度等改革。深化财税体制改革。认真落实新《预算法》，完善政府预算体系，将政府收支全部纳入预算管理；加强财政绩效管理，规范政府债务管理；大力压缩财政一般性支出，确保重点领域和民生支出。深化投融资体制改革。大力推行政府与社会资本合作（PPP）模式，吸引社会资本参与基础设施、产业发展、社会事业等重点领域建设；健全完善投融资平台公司管理机制；深入推进土地、矿业权抵押融资。深化开发区与园区体制机制改革。推进临汾、侯马开发区扩区，做好襄汾一区两园和洪洞赵城煤焦化深加工园区申报省级开发区工作。同时，积极推进政府机构、国有企业、工商登记制度、户籍制度等重点领域改革，进一步扩大对外贸易，提高对外开放水平。

（四）全力推动三个突破，有效破解制约瓶颈全力推进科技创新。加强与中科院北京分院的战略合作，加快实施创新驱动战略和低碳创新行动计划。强化企业技术创新主体地位，提升企业自主创新能力，推动高新技术产业发展，重点培育10个优势产业集群。以安全、清洁、高效、低碳发展为方向，实施好煤基科技重大专项，支持7条煤基产业创新链建设。加快科技研发平台建设，省级以上工程（技术）研发中心、企业技术中心达到19家以上。出台《临汾市科技人才“市长创新奖”工作方案》和《关于建立完善科研人员股权和分红激励政策的实施意见》，积极引进高端人才和创新团队。加强知识产权管理，全市有效发明专利拥有量增长10%。

大力发展民营经济。认真落实促进民营经济发展的政策措施，进一步放宽市场准入，设立临汾市民营经济发展基金，努力为民营企业创造公平竞争的市场环境。积极搭建创业平台，大力培育各类创业主体，鼓励支持企业家“二次创业”，继续推进小微企业创办、“小升规”企业成长、“小巨人”企业提升等工程，新培育“小巨人”企业8户,“小升规”企业23户，创办小微企业2000户。制定政策，搭建平台，拓展空间，营造氛围，加快形成大众创业、万众创新的生动局面。

切实推动金融振兴。加大金融机构引进力度，加快村镇银行、社区银行和小微银行建设步伐。培育壮大资本市场，完善短期融资券、中期票据、中小企业集合债券等融资工具，加快企业上市步伐。围绕服务实体经济，完善政银企合作机制，促进信贷融资、债券融资、股权融资和上市融资全面发力。加快发展现代保险服务业。加强诚信体系建设。优化金融生态，防范和打击非法集资。

（五）扎实做好“三农”工作，促进农业发展农民增收夯实农业发展基础。加强农田水利基本建设，加大农机新技术、新机具推广力度。坚持把保障粮食安全作为首要任务，严守耕地红线，确保耕地面积稳定在700万亩以上。扎实推进粮食创高产工程，重点建设31个万亩高产示范区，推广标准化高产高效、绿色环保技术模式，确保全市粮食总产稳定在20亿公斤以上。

发展特色现代农业。按照产出高效、产品安全、环境友好的要求，推进“四个百万亩”特色农业基地建设，发展精细蔬菜5万亩、水果5万亩、中药材5万亩、以核桃为主的干果经济林10万亩。加快现代农业园区建设，提高农业规模化、集约化、现代化水平。支持培育种养大户、家庭农场、农民专业合作社、农业企业等新型经营主体，积极发展多种形式的适度规模经营。继续抓好“两平台一通道”建设，巩固完善市、县、乡、村农业科技服务体系，扶持发展休闲农业、观光农业。大力开展农产品质量安全专项整治，确保“餐桌上的安全”。

持续增加农民收入。把农民增收摆在突出位置，认真落实好种粮直补、农资综合直补、良种补贴等各项强农惠农政策。鼓励引导发展规模种养、农产品加工。强化农民技能培训，全年培训新型职业农民1.5万名，促进农村富余劳动力转移就业。

扎实推进扶贫开发。按照精准扶贫的要求，大力实施产业扶贫开发工程，完成投资22亿元，重点推进5个国定贫困县与4家省属企业的扶贫项目建设。推进国家光伏扶贫试点，光伏发电试点村总数达到100个、装机总容量达到10兆瓦。完成9个贫困县、1.2万人的易地搬迁任务。做好大宁、吉县连片特困地区区域发展与扶贫攻坚试点工作。抓好干部驻村帮扶、定点扶贫、金融扶贫、整村推进和外资扶贫等各项扶贫开发重点工程。实现6.45万贫困人口脱贫，减少60个贫困村，确保贫困地区农村居民人均可支配收入增幅高于平均水平。

（六）统筹城乡一体发展，提高城镇化质量和水平积极稳妥推进城镇化。完成《临汾市城市总体规划（2009-2020）》评估，编制《临汾市城市地下管线综合规划》、《汾河景区控制性详细规划》等规划，市区控规覆盖率达到100%，各县市控规覆盖率达到60%以上。围绕“一带两圈多点”的城镇化布局，扎实推进汾河沿线城镇带、临汾都市圈和侯马都市圈、12个大县城和60个重点镇建设。

加快推进农业转移人口市民化，力争全市城镇化率达到49%以上，汾河沿线城镇带内城镇化率达到53%以上。启动实施城市人居环境改善工程。编制《城市人居环境改善工程三年规划》，全面实施设施提升、城市安居、城中村改造、环境提质四大工程。

加快中心城市建设步伐。按照“拉大框架、完善功能、提升品位、宜居宜业”的要求，加快市区建设步伐，提升城市综合承载能力。完善提升交通路网，构建“八横十纵两环”的城市交通体系。改造3条老旧路，对鼓楼南街、煤化巷至鼓楼东大街步行街、锣鼓大桥西延进行拓宽改造；打通3条断头路，加快中大街南延工程建设，启动华州路东延、迎春街南延等工程；建设一批主干路，完成规划三街南段、漪汾花园南路、西赵路西段、中大街南段、市中心地震台应急通道等道路工程，启动规划三街北段、西赵路东段等道路建设，对滨河东路、迎宾大道等重要交通节点实施立交快速化改造，建设市区通往机场的快速通道。加快城中村和棚户区改造步伐，实施尧都西关社区、盘龙社区、尧庙镇郭村、刘村镇涧头村等城中村改造，新开工城镇保障房7424套，基本建成14800套。完成北城壕西段防汛排水及道路工程，启动向阳西路、秦蜀路南延、鼓楼西街西段、机场大道等排水工程。加快污泥处置项目、龙祠水源净水厂改扩建、市应急水源地等工程建设。推进市图书馆、博物馆、奥体中心、市戏剧研究院非遗保护传承基地等项目建设，提升城市综合品位和整体形象。

持续抓好农村人居环境改善。实施好农村人居环境改善四大工程，高标准建设园林村60个，新建农村老年人日间照料中心80个，实施广播电视卫星户户通3万户；加快采煤沉陷区治理，搬迁安置群众2887户、9843人；完成1192户受地质灾害威胁严重的农民群众搬迁任务；实施农村危房改造9790户；推进35个农村污水防治试点村、100个畜禽粪污治理和100个病死动物无害化处理示范点建设；扎实开展美丽宜居示范村“三级联创”活动，重点抓好20个省级、30个市级美丽宜居示范村的创建工作，促进农村环境全面提升。

（七）抓好社会治理创新，维护社会和谐稳定持之以恒抓好安全生产。牢固树立安全生产“红线”意识和安全发展理念，严格落实新《安全生产法》，实现安全监管责任“五个全覆盖”。采用“四不两直”以及明察暗访等方式，深入开展安全生产大检查和打非治违专项行动，全面加强煤矿、非煤矿山、道路交通、油气管道、尾矿库、水库、食品药品、危险化学品、民爆物品、烟花爆竹、人员密集场所、消防、学校、校车、建筑施工、森林防火、特种设备等各行业、各领域的安全生产工作。严格落实安全生产目标责任考核“一票否决制”，确保全市安全生产形势持续明显好转。

积极推动社会治理创新。推进网格化管理和基层综合服务管理平台建设。落实信访工作责任制和重点案件领导包案制度，建立畅通、规范、有序的群众诉求表达渠道。完善人民调解、行政调解、司法调解“三调联动”大调解工作机制，确保绝大多数矛盾纠纷在基层得到有效化解。深入推进“平安临汾”“法治临汾”建设，持续开展打黑除恶专项行动，完成市区新建整合视频监控工程建设，开展以“城中村”为重点的治安整治，加大反恐工作力度，提高群众的安全感和满意度。强化应急救援管理，积极推进政府各部门应急处置信息共享机制，妥善处理各类突发事件。加强特殊人群管控矫治，确保社会和谐稳定。

（八）加强生态文明建设，持续改善生态环境抓好节能减排工作。完善节能减排工作制度和推进措施，统筹做好工业、建筑、交通等重点领域节能减排工作。全力实施好“国家节能减排财政政策综合示范城市”项目，重点推进城市环境整治及企业污染物减排、交通清洁化、绿色建筑、余热余压循环利用、产业低碳化等五大类45个典型示范项目。扎实做好能耗强度控制、节能评估和审查等工作，万元地区生产总值能耗下降4.29%，确保超额完成“十二五”节能减排目标。

加大环境保护力度。围绕创建国家环保模范城目标，认真贯彻落实新《环保法》，全力打好大气、水、土壤污染治理三大战役。扎实推进大气污染防治五年行动计划，组织开展工业企业污染治理、煤烟污染治理、建筑施工工地扬尘治理、道路扬尘治理、机动车污染治理和油气治理等6项大气污染防治工程，市区完成天然气改造5.7万户，城中村、城郊村燃煤锅炉改造1100户，新增供热面积120万平方米，全面完成黄标车淘汰任务，确保PM2.5浓度稳步下降。继续实施地表水跨界断面考核全覆盖，扎实开展河流清洁行动。加快市区河西生活污水处理厂、城北污水处理厂推进力度。抓好龙祠等城市饮用水水源地的综合治理和监管。持续加大土壤污染防治工作力度，开展土壤污染调查及治理试点工作。

提升生态建设水平。按照林业建设“三加三不减”的要求，以“两山、两网、两林、两区、双保”五大国家、省级造林工程为龙头，重点实施好国家新一轮退耕还林、霍永高速绿化、吕梁山生态脆弱区植被恢复等工程，完成造林39.57万亩，确保森林覆盖率增加1个百分点。按照国家主体功能区定位，在西山7县实施生态试点示范区、特色生态农业发展、黄河旅游带建设、生态综合开发、绿色工业产业基地建设等五大行动计划，建立完善因地制宜、绿色发展的生态经济体系。市区创建国家级园林城市。

（九）全面发展社会事业，着力保障和改善民生大力加强文化建设。实施“文化强市”战略，大力弘扬廉政文化、红色文化、法治文化，深入挖掘我市优秀传统历史文化。办好山西?临汾帝尧古都文化旅游节，加快建设市“五馆一院”、县“三馆一院”和乡镇文化站所、农村文化活动场所。建立文化产业项目精品库，推进“县级城市数字影院建设工程”。完成广播电视“户户通”2万户。加大历史文物和非物质文化遗产保护力度，建立以非物质文化遗产为主的生态保护实验区。积极筹备第十七届“群星奖”比赛，开展好第三届梨花奖评比活动。强化文化服务，坚持文化下乡。举办临汾市第四届运动会。

做好扫黄打非和净化网络工作。优先发展教育事业。扎实推进义务教育办学模式改革，全面改善义务教育薄弱学校和寄宿制学校基本办学条件。启动中高等院校资源整合工作，推进山西师大、师大文理学院和临汾学院新校区建设。持续提升医疗卫生水平。打造以“三甲”医院为龙头的医疗联合体，加强公共卫生服务，为农村60岁以上老年人实行免费体检。开展国家级和省级卫生城市（县城）创建活动。坚持计划生育基本国策，人口自然增长率控制在6.5‰以内。不断提高社会保障水平。统筹推进城乡社保体系建设，提高企业退休人员基本养老金、城乡居民基础养老金、城镇职工医保最高支付限额、工伤保险等标准。大力发展养老福利事业，推进养老服务社会化、专业化。

在做好上述工作的同时，我们还要高标准编制国民经济和社会发展第十三个五年规划；深入实施军民融合发展战略，支持驻临部队、武警部队、民兵预备役和人防建设；支持工会、妇联、共青团等人民团体工作；认真做好审计、统计、档案、气象、水文、地震、民族宗教、外事侨务、对台、老龄和残疾人等各项工作。

各位代表，今年，我们要继续实施“六大惠民工程”。

实施学前教育优质普惠工程。深入实施第二期学前教育三年行动计划，新改扩建标准化公办幼儿园28所、农村幼儿园36所。严格落实城区幼儿园建设规划，加快城镇住宅小区配套幼儿园建设。继续清理规范民办幼儿园，探索以政府购买服务的方式发展学前教育，学前三年入园率达到80%以上。

实施乡村医疗服务提质工程。继续实施基本药物制度，做好常用低价药品供应保障工作，实施基层中医药服务能力提升工程。巩固“新农合”制度，人均筹资标准由390元提高到470元，确保参合率稳定在95%以上。

实施农业强基工程。加强农田水利基本建设，扎实推进我市“小水网”建设，加快西山沿黄提灌工程，完成引沁入汾浮山供水工程输水干线10.56公里建设。投资1.54亿元，重点实施6个县的中央财政小型农田水利重点县项目建设，全年完成实灌面积231.4万亩。

实施饮水安全工程。加大农村饮水工程建设力度，提高和改善7.9万农村人口和乡镇学校师生的饮水安全标准。完成县级水质检测中心建设任务。新建和改造城市供水管网70公里，供水普及率达到96.5%。

加快城乡供水一体化步伐。

实施城市综合整治工程。开展环境秩序、交通秩序、临街门面、违章建设等四项专项治理行动，切实解决“马路市场”、暴露性垃圾堆积、黑出租（三轮）非法营运、车辆乱停乱放、户外广告牌匾违章设置、乱搭乱建等问题，提升城市精细化管理水平。落实公交优先战略，全市更新城市公交车100辆。实施“放心早餐工程”，解决城区早餐市场“脏、乱、差”问题。建设垃圾处理产业园区，启动实施垃圾焚烧发电和餐厨垃圾处理等项目。

实施就业创业援助工程。全方位落实就业扶持措施，积极开发就业见习、三支一扶和基层公益性岗位。大力实施大学生创业引领计划，开展以创业带动就业工作。全市城镇新增就业5.1万人，转移农村劳动力就业5.1万人，城镇登记失业率控制在4%以内。

三、切实加强政府自身建设做好今年的各项工作，必须进一步加强政府自身建设，不断提高执政能力和工作水平。

强化权力监督。把坚持“六权治本”作为加强政府自身建设的根本要求，贯彻落实到政府工作的各个方面。依法确定权力，做到职权法定、简政放权；科学配置权力，实现制约权力和效率、效能的统一；制度约束权力，按照“全面覆盖、全程到位、制度束权”要求，完备制度设计，优化制度运行，严格制度执行；阳光行使权力，坚持以公开为常态，全面落实公开制度，确保公开实效；合力监督权力，坚持全方位、全领域、全过程监督权力，切实提高监督效能；严惩滥用权力，严厉查处“乱作为”，严肃问责“不作为”。自觉接受市人大及其常委会的法律监督、工作监督和市政协的民主监督，认真办理人大代表建议和政协委员提案，广泛听取各民主党派、工商联、无党派人士和人民团体的意见建议。加强审计监督，大力推进公共资金、国有资产和领导干部履行经济责任审计全覆盖。按照《临汾市政府信息公开暂行办法》的有关规定，加大政务信息公开力度，全面接受舆论和社会监督。

坚持依法行政。深入贯彻党的十八届四中全会精神，认真落实《关于加快推进法治临汾的实施意见》，把法治理念和法治思维贯穿政府工作全过程，做到法定职责必须为、法无授权不可为。不断完善《市政府议事规则》，建立政府法律顾问和智库制度，健全行政重大决策的法定程序、合法性审查机制、责任追究制度，推进决策科学化民主化。深化行政综合执法体制改革，加强行政执法队伍建设，做到严格规范公正文明执法。

改进工作作风。巩固党的群众路线教育实践活动和学习讨论落实活动成果，持续整治“四风”，健全改进作风常态化制度。

认真开展“三严三实”专题教育，着眼于坚定理想信念、强化党性观念、增强实干精神，着力解决“不严不实”的突出问题，确保专题教育取得实实在在的成果。从严管理公务员队伍，强化实干意识、担当意识、执行意识，加大督查问责力度，形成干事创业的良好氛围。密切联系群众，畅通市长热线，落实领导干部下基层工作机制，有效解决服务群众“最后一公里”问题。

加强廉政建设。严格落实党风廉政建设责任制和党委（党组）的主体责任，加强惩治和预防腐败体系建设，用制度管权、管钱、管人，形成不敢腐的惩戒机制、不能腐的防范机制、不想腐的保障机制。认真执行“八项规定”、“约法三章”，严控“三公”经费。

推进机关公务用车制度改革。加大专项治理力度，按照全市《专项整治实施方案》要求，认真开展涉及政府系统的31项专项整治，完善重点部门和关键岗位的廉政风险防控措施，树立为民务实清廉的良好形象。

各位代表，新常态孕育新机遇，新机遇催生新发展。让我们更加紧密地团结在以习近平同志为总书记的党中央周围，在市委的坚强领导下，在市人大、市政协的监督支持下，团结和带领全市人民，真抓实干，开拓创新，全面完成今年经济社会发展和“十二五”规划各项目标任务，为开创弊革风清、富民强市新局面而努力奋斗！
